# Sidelobe-free deterministic 3D nanoscopy with λ/33 axial resolution

**DOI:** 10.1038/s41377-025-01833-x

**Published:** 2025-04-21

**Authors:** Binxiong Pan, Baoju Wang, Yue Ni, Qi Zhao, Yuqi Wang, Yuyan Cai, Qiuqiang Zhan

**Affiliations:** 1 Centre for Optical and Electromagnetic Research & Guangdong Engineering Research Centre of Optoelectronic Intelligent Information Perception, South China Academy of Advanced Optoelectronics, South China Normal University, Guangzhou, China; 2 National Center for International Research on Green Optoelectronics & Guangdong Provincial Key Laboratory of Optical Information Materials and Technology, South China Academy of Advanced Optoelectronics, South China Normal University, Guangzhou, China; 3 MOE Key Laboratory & Guangdong Provincial Key Laboratory of Laser Life Science, South China Normal University, Guangzhou, China

**Keywords:** Nanoparticles, Super-resolution microscopy, Biophotonics, Nonlinear optics

## Abstract

Deterministic three-dimensional (3D) super-resolution microscopy can achieve light-matter interaction in a small volume, but usually with the axial extension distinctly more elongated than the lateral one. The isoSTED method combining two opposing objectives and multiple laser beams can offer high axial extension at λ/12 level, but at the cost of optical system complexity and inherent sidelobes. The high-order nonlinear effect by multiphoton excitation would benefit to achieve a sub-diffraction resolution as well as to suppress the sidelobes. Herein, to achieve an easy-to-use, sidelobe-free deterministic 3D nanoscopy with high axial resolution, we developed a purely physical deterministic strategy (UNEx-4Pi) by fusion of ultrahighly nonlinear excitation (UNEx) of photon avalanching nanoparticles and mirror-based bifocal vector field modulation (4Pi). The theoretical studies of UNEx-4Pi concept showed that the main peak of fluorescence spot became sharper and its large sidelobe height was suppressed with the increasing optical nonlinearity. In addition, the simplicity and robustness of UNEx-4Pi system were demonstrated utilizing a mirror-assisted single-objective bifocal self-interference strategy. Experimentally, UNEx-4Pi realized an extremely constringent focal spot without sidelobes observed, achieving an axial resolution up to λ/33 (26 nm) using one low-power CW beam. We also demonstrated the super-resolution ability of the UNEx-4Pi scheme to bioimaging and nuclear envelope of BSC-1 cells were stained and imaged at an axial resolution of 32 nm. The proposed UNEx-4Pi method will pave the way for achieving light-matter interaction in a highly confined space, thereby advancing cutting-edge technologies like deterministic super-resolution sensing, imaging, lithography, and data storage.

## Abstract (web summary)

A purely physical, single objective-lens strategy (UNEx-4Pi) for achieving sidelobe-free λ/33 axial resolution by fusion of ultrahighly nonlinear excitation of photon avalanches and mirror-assisted bifocal vector field modulation.

## Graphical abstract

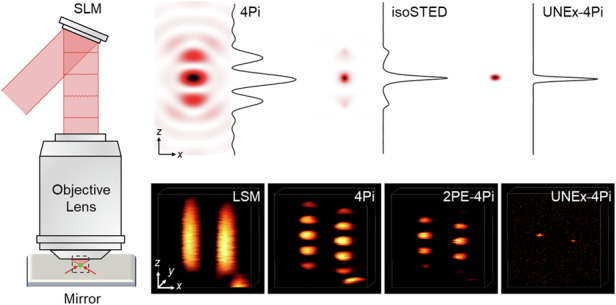

## Introduction

Enabling the study of light-matter interaction in a three-dimensionally and highly confined space is of utmost importance, but also very challenging for many physics studies and frontier technologies, such as diffraction-unlimited imaging^1^, sensing^2,3^, lithography^4,5^, data storage^6,7^, and particle manipulation^8^. For instance, various deterministic or stochastic three-dimensional (3D) microscopy modalities have been developed as powerful tools for super-resolution imaging by breaking the diffraction limit through optically-controlled fluorescence processes^9–17^. Utilizing the interference of three beams, 3D structured illumination microscopy usually offers λ/4 lateral and λ/2 axial resolutions^18^. The stochastic 3D single-molecule localization microscopy (SMLM) can provide λ/20 lateral and λ/8 axial resolutions by localizing photo-switchable fluorescent probes^19^. 3D stimulated emission depletion (STED) microscopy enables superior resolution of λ/10 laterally and λ/4 axially by adding two high-power phase-modulated depletion beams^20^. Minimal photon fluxes (MINFLUX) microscopy can enable ultra-high spatial resolution of 1–3 nm by incorporating two phase-modulated beams and photoswitching-based single-molecule localization reconstruction^21–23^, but at the cost of time resolution and relies on highly complicated hardware and reconstruction algorithms. To date, most of 3D nanoscopy modalities are subjected to an axial resolution two or three times worse than the lateral one, since only a small fraction of the uniform spherical wavefront can be collected by an objective lens.

**Fig. 1:**
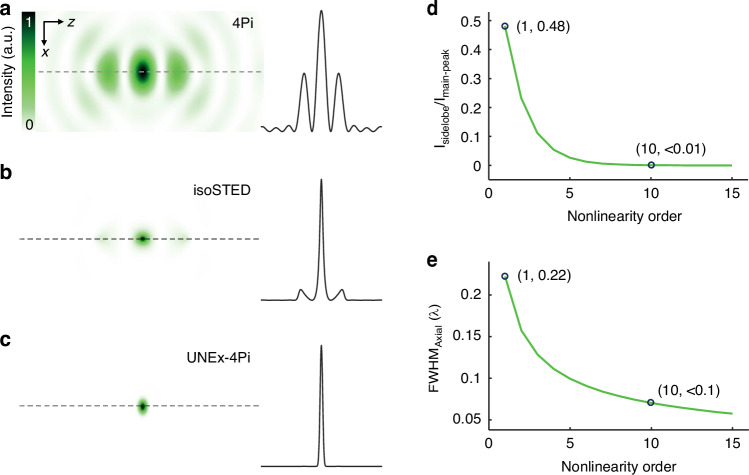
The sidelobe effect in 3D microscopy. **a–c** The simulated focal intensity distributions of 4Pi, isoSTED and UNEx-4Pi. The plots on the right showed the intensity profiles along the dash lines. **d** The suppressing of sidelobes (intensity ratio *I*_*sidelobe*_/*I*_*main-peak*_) versus the fluorescence nonlinearity order in multiphoton 4Pi microscopy. **e** The narrowing axial FWHM of PSF with the increasing nonlinearity order in 4Pi microscopy

To improve the axial confinement, the 4Pi microscopy was developed to coherently add the spherical wavefronts of two opposing objective lenses and compress the axial point spread function (PSF) (Fig. 1a)^24–26^. Furthermore, the combination of 4Pi and STED, namely isoSTED^27^, can obtain a nearly isotropic focal spot of λ/12 in three dimensions (Fig. 1b). However, isoSTED nanoscopy suffers from practical limitations due to the high complexity of optical system with six laser beams and double objective lenses. Although a 4Pi focus can also be generated by a setup of a mirror and one single objective lens^28^, the intrinsic interference nature gives rise to a train of high-energy sidelobes accompanying the main lobe for all 4Pi and its derivative techniques, thereby spreading the light-matter interaction i.e., degrading the imaging contrast and inducing photobleaching (Table S1). In practice, the refractive index mismatch between the silicone oil and the mounting medium would lead to depth-dependent aberrations, and thus depth-dependent sidelobes^29^. The sample thickness of 4Pi microscopes is typically limited to 10 μm^30^. Furthermore, the imperfect alignment of dual objectives further aggravates the sidelobe problem. In previous studies, the sidelobe height can exceed 60% of the main peak in Type A 4Pi (only excitation wavefronts interfere) microscopy^31^, even approaching 70% in non-confocal detection mode^32^. Two-photon excitation (2PE) can reduce the sidelobe height by a factor of four thanks to the large difference in excitation and detection wavelengths^33^. The combination of 2PE and confocal detection in Type C 4Pi (both excitation and detection wavefronts interfere) microscopy further reduces the sidelobe height to 5% and significantly enhances image contrast^34^. However, 2PE microscopy has inherently a reduced resolution owing to its doubled excitation wavelength. Another approach to suppress these adverse sidelobes is to perform mathematical deconvolution on the 3D raw image data^35–37^. Nevertheless, the prerequisites for high-performance deconvolution treatment are high signal-to-noise ratio (SNR) images and accurate priori parameters estimation, which will take a lot of computation time and may introduce additional artifacts. Importantly, deconvolution processing only eliminates the sidelobe effect within the virtual image space, that is, in the real space such sidelobes still keep exerting influence on light-matter interactions. Therefore, the complete extinction of sidelobes in real physical space for improving 4Pi microscopy is of utmost importance, but remains an unsolved stubborn problem.

Lanthanide-doped upconversion nanoparticles (UCNPs) have been utilized in sub-diffraction multiphoton microscopy imaging^38,39^ due to their advantageous properties, including high optical nonlinearity (*N*), anti-Stokes shifts, and resistance to photobleaching. As a unique upconversion process, photon avalanche (PA) exhibits an exceptionally strong nonlinear optical response^40–42^, thereby enabling PA nanoscopy to achieve resolutions of ~60 nm laterally and ~180 nm axially in a standard laser scanning microscope (LSM)^43^. In addition, the giant PA nonlinearity holds considerable promise in eliminating the undesirable sidelobes in 4Pi microscopy. In this work, we developed a purely physical, deterministic strategy, namely UNEx-4Pi, by combining ultrahighly nonlinear excitation (UNEx) of PA nanoparticles and mirror-based bifocal vector field modulation (4Pi). The theoretical analysis demonstrated that high-order optical nonlinearity (*N* > 10) can completely eliminate the sidelobes (height < 0.1%) with no need of any mathematical deconvolution postprocessing (Fig. 1c). Experimentally, PA nanoprobes were fabricated and exhibited huge optical nonlinearity up to 35th-order, and UNEx-4Pi realized an extremely constringent fluorescence excitation spot (26 nm (λ/33) in axial direction) without any observed sidelobe (zero). We further demonstrated UNEx-4Pi super-resolution bioimaging for labeled cells and achieved an axial resolution of 32 nm.

## Results

### Theory modelling and simulation for sidelobe-free UNEx-4Pi nanoscopy

To verify the efficacy of ultrahighly nonlinear excitation (UNEx) on eliminating the high-intensity sidelobes in 4Pi microscopy, we firstly performed theory modelling and simulation. A linearly polarized electric field distribution in objective space was defined based on the Wolf vector diffraction theory^44^:

1 $${\rm{E}}(\rho ,\phi ,{\rm{z}})=({{\rm{E}}}_{x}{,{\rm{E}}}_{y}{,{\rm{E}}}_{z})=-{\rm{i}}C({I}_{0}+{I}_{2}\,\cos (2\phi ),{I}_{2}\,\sin (2\phi ),-2i{I}_{1}\,\cos \phi )$$

2 $$\begin{array}{ll}{I}_{0}=\mathop{\int }\limits_{0}^{\alpha }\sqrt{\cos \theta }\sin \theta (1+\,\cos \theta ){J}_{0}({k}_{1}\rho \,\sin \theta ){e}^{i{k}_{1}z\cos \theta }d\theta \\ {I}_{1}=\mathop{\int }\limits_{0}^{\alpha }\sqrt{\cos \theta }{\sin }^{2}\theta {J}_{1}({k}_{1}\rho \,\sin \theta ){e}^{i{k}_{1}z\cos \theta }d\theta \\ {I}_{2}=\mathop{\int }\limits_{0}^{\alpha }\sqrt{\cos \theta }\sin \theta (1-\,\cos \theta ){J}_{2}({k}_{1}\rho \,\sin \theta ){e}^{i{k}_{1}z\cos \theta }d\theta \end{array}$$

where $(\rho ,\phi ,{\rm{z}})$ is cylindrical coordinate, *C* is a normalized constant, *k*_*1*_ is the wavenumber in medium, $\alpha$ is the half-aperture angle of the objective lens, and $\theta$ represents the polar angle. ${J}_{n}$ are Bessel functions of the first kind. For a point scanning (non-confocal) arrangement, the PSF is governed by the excitation light. Hence, the PSF of 4Pi (Type A) can be expressed as:

3 $$\begin{array}{l}PS{F}_{4Pi}={|E(\rho ,\phi ,{\rm{z}})+E(\rho ,\phi ,-{\rm{z}})|}^{2}\\\qquad\quad\;\;=4{C}^{2}\left({\mathrm{Re}}^{2}\{{I}_{0}\}+2\mathrm{Re}\{{I}_{0}\}\mathrm{Re}\{{I}_{2}\}\cos 2\phi\right.\\\qquad\quad\;\;+\,\left.{\mathrm{Re}}^{2}\{{I}_{2}\}+4{\text{Im}}^{2}\{{I}_{1}\}{\cos }^{2}\phi \right)\end{array}$$

Given the symmetric distribution of the PSF along the optical axis, considering only the intensity distribution on the optical axis (*ρ* = 0) allows for the omission of *J*_*1*_ and *J*_*2*_ in formula (3), thereby simplifying it further as follows:

4 $$PS{F}_{4Pi}(z)={{\rm{Re}}\{{\rm{I}}_{0}\}}^{2}=\left\{{\int }_{\cos \alpha }^{1}\sqrt{u}(1+u)\cos ({k}_{1}zu)du\right\}^{2}$$

By incorporating the multiphoton effect (nonlinearity order, *N*) into 4Pi illumination, the PSF can be rewritten as:

5 $$PS{F}_{multiphoton\_4Pi}(z)={\{PS{F}_{4Pi}(z)\}}^{N}={\left\{{\int }_{\cos \alpha }^{1}\sqrt{u}(1+u)\cos ({k}_{1}zu)du\right\}}^{2N}$$

The intensity ratio and the energy ratio can then be derived to quantify the nonlinear effects on sidelobes:

6 $${I}_{sidelobe}/{I}_{main-peak}=PS{F}_{multiphoton\_4Pi}({z}_{1})/PS{F}_{multiphoton\_4Pi}(0)$$

7 $${E}_{main-peak}/{E}_{total}={\int }_{-{z}_{2}}^{{z}_{2}}PS{F}_{multiphoton\_4Pi}(z)dz/{\int }_{-\infty }^{+\infty }PS{F}_{multiphoton\_4Pi}(z)dz$$

*I*_*sidelobe*_ and *I*_*main-peak*_ are the peak intensities of the primary sidelobe and the main peak, respectively, *E*_*main-peak*_ is the integral energy of the main peak, *E*_*total*_ is the integral energy of the total spot, *z*_*1*_ is the position of the primary sidelobe, and *z*_*2*_ is the position of the minima between the main peak and the sidelobe. Low-order nonlinearity of traditional multiphoton excitation cannot fundamentally overcome the severe sidelobes of the PSF_4Pi_, which can be completely eliminated only when the nonlinearity order *N* reached 10 (Figs. 1d and S1). In addition, the axial full width at half maximum (FWHM) of the main focal spot in 4Pi illumination is given by $dz\approx \lambda /3n$ in the focal plane, with *λ*, *n* denoting the wavelength and the refractive index, respectively^45^. In the context of multiphoton excitation, it is rewritten as $dz\approx \lambda /3n\sqrt{N}$, meaning that the resolution gets improved with the nonlinearity (Fig. 1e).

**Fig. 2:**
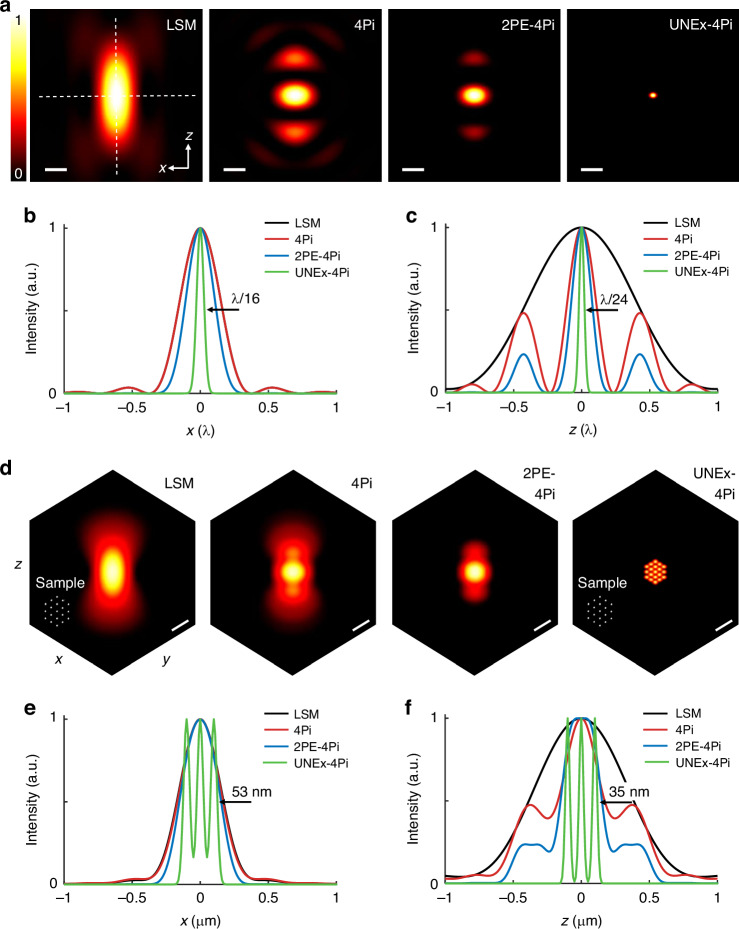
Simulation of PSFs for different excitation methods. **a** The *xz*-sections of the intensity distributions in LSM, 4Pi, 2PE-4Pi, and UNEx-4Pi excitation modes. Scale bar: λ/4. λ is the excitation wavelength, NA of the objective lens is 1.45. **b**, **c** showed the corresponding lateral and axial intensity profiles of (**a**). The lateral FWHM of UNEx-4Pi is λ/16, and the axial FWHM is λ/24. **d** The simulated results of different excitation modes for a 3 × 3 × 3 sample array with a space interval of 100 nm. Scale bars: 400 nm. **e**, **f** showed the corresponding lateral and axial intensity profiles of (**d**)

Based on the above theoretical modelling, the effective PSFs of laser scanning microscopy (LSM), 4Pi, 2PE-4Pi and UNEx-4Pi modes were simulated, respectively. As shown in Fig. 2a, with a fluorescence nonlinearity order of 30, UNEx-4Pi completely eliminated the sidelobes in 4Pi or 2PE-4Pi modes. Meanwhile, the ultrahigh nonlinearity is beneficial to shrink the size of PSF^41^. The effective PSF volume of UNEx-4Pi was only 1/500 of that in LSM, exhibiting lateral and axial FWHM of λ/16 and λ/24, respectively (Fig. 2b, c). In contrast, in conventional LSM mode the UNEx effect can only provide an axial FWHM of λ/6, which is still 2–3 times worse than the lateral one (Fig. S2). A designed nanoparticles array with a separation interval of 100 nm was used to investigate the excellent 3D resolution of UNEx-4Pi (Fig. 2d). Here, the excitation wavelength (λ) was set to 852 nm. The imaging resolution of traditional LSM was constrained by the diffraction-limit, missing the fine structural information, particularly along the optical axis. Although the 4Pi microscopy compressed the axial PSF size to some extent, the sidelobes resulting from light interreference seriously blurred the obtained image. Similarly, in 2PE-4Pi microscopy the effect of two-photon excitation can further weaken the sidelobes, but the limited resolution and the residual-sidelobes induced artifacts made it yet unable to distinguish sub-100 nm fine structures. In contrast, UNEx-4Pi can clearly distinguish each adjacent points, with simulated lateral and axial FWHMs of 53 nm and 35 nm, respectively (Fig. 2e, f).

### High robustness and high stability of the UNEx-4Pi system

**Fig. 3:**
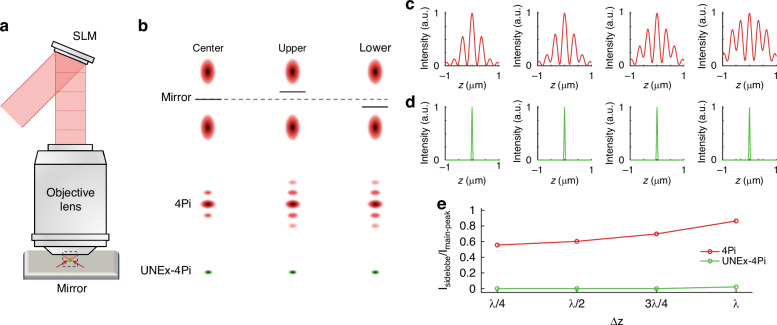
High robustness and high stability of the UNEx-4Pi system. **a** Schematic diagram of a mirror-based single-objective 4Pi setup. **b** The sidelobe effect diagram for the misalignment of the two foci in 4Pi and UNEx-4Pi modes. The dash line represented the centre position of the foci, and the solid line represented the position of the mirror. **c** Intensity profiles of the simulated PSF_4Pi_ along the *z*-axis. From left to right, the deviation Δz were λ/4, λ/2, 3λ/4, and λ, respectively. Δz represents the centre-to-centre deviation of the two foci after the specular reflection. **d** Intensity profiles of the simulated UNEx-PSF_4Pi_ (*N* = 30), corresponding to (**c**). **e** The plot of intensity ratio *I*_*sidelobe*_/*I*_*main-peak*_ versus Δz

As previously discussed, 4Pi microscopy necessitates precise alignments of two opposing objectives and multiple optical paths, which is a challenging task requiring high costs, hourly calibrations and painstaking maintenance (Fig. S3). To simplify this conventional dual-objective 4Pi system, a mirror was introduced to serve as a substitute for one of the objective lenses (Fig. 3a). The essence of a single-objective 4Pi scheme is the generation of two separated focal spots along the optical axis via vector optical field modulation. To generate two spots at *r*_*0*_ and −*r*_*0*_ with respect to the focal plane, the incident beam is shaped based on the principles of time-reversal focusing theory^46^. Specifically, the pattern displayed on the spatial light modulator (SLM) is defined by the following equation^47^:

8 $$\varphi (\theta )=\frac{\pi }{2}sign[\sin ({r}_{0}{k}_{1}\,\cos \theta )]$$

Subsequently, a mirror for specular reflection was placed at the center of the two foci to induce the interference. Considering the specular reflectance may lead to intensity discrepancies between the two foci, the impact of this factor was studied (Fig. S4). The simulations showed that the intensity discrepancies can be neglected for the PSF_4Pi_ as long as the mirror reflectance exceeded 80%. In practice, the reflectance of commercial mirrors is generally larger than 90%. Therefore, the focus produced by this mirror-assisted single-objective self-interference is equivalent to that of a dual-objective Type A 4Pi system.

Achieving effective 4Pi focusing necessitates the precise alignment of two excitation spots along the optical axis, thereby the position of the mirror is critical for generating effective interference between the two foci. An inaccurate mirror position can lead to a centre-to-centre deviation (Δz) of the two foci, which would exacerbate the sidelobe problem (Fig. 3b). It was calculated that the intensity of the primary sidelobe was maintained at 50% of the main peak, when the foci deviation was λ/4. Further, as the foci deviation increased to λ, the sidelobe intensity increased significantly to 80% of the main peak (Fig. 3c). As such, for a linear fluorescence process, the required foci deviation for not increasing the sidelobe intensity must be smaller than 100 nm. As a stark contrast, UNEx-4Pi exhibited a remarkably high tolerance to this foci deviation due to the ultrahigh-order fluorescence nonlinearity (Fig. 3d). UNEx-4Pi could consistently maintain a compact, sidelobe-free PSF within a foci deviation of up to one wavelength. In other words, the UNEx-4Pi setup was insensitive to the foci deviation in a large range of 850 nm, featuring high system robustness (Fig. 3e). Compared with conventional 4Pi microscopy, UNEx-4Pi would not only reduce the system complexity, but also relieve the burdensome system calibrations greatly (Fig. S5), which is highly conducive to the promotion of 4Pi illumination technology.

### The dynamic scanning range of UNEx-4Pi nanoscopy

**Fig. 4:**
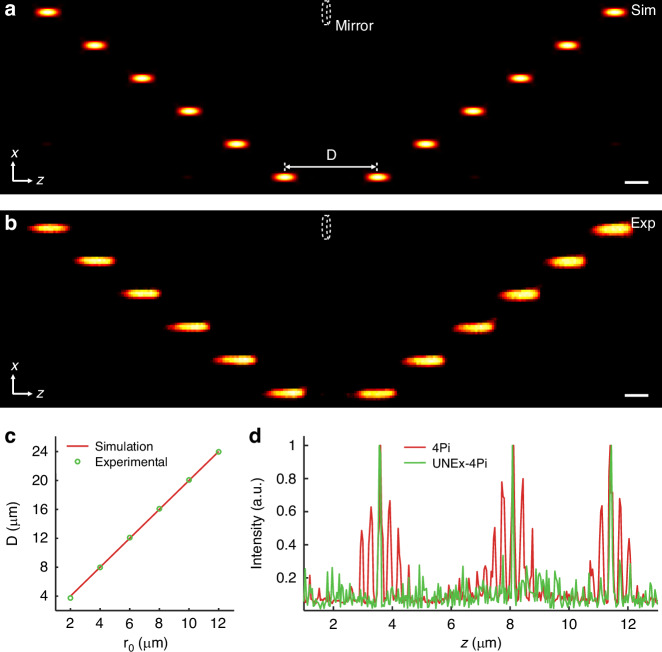
Axial dynamic scanning range of deterministic UNEx-4Pi. **a** The pairs of excitation spots were generated by different parameters (*r*_*0*_ = 2, 4, 6, 8, 10, and 12 μm) in the absence of the mirror based on phase-modulated simulation. The distance between the centers of the two foci, designated as D, was determined through a Gaussian fit. **b** Experimental fluorescence images of a single nanoparticle in the absence of the mirror, consistent with the simulation results. **c** The linear correlation between D and *r*_*0*_ indicated D = 2*r*_*0*_. **d** Intensity profiles of fluorescence images of PA nanoparticles along the axis were obtained at 3.6 μm, 8.1 μm, and 11.4 μm from the mirror surface, respectively. Scale bars: 1 μm

To experimentally validate that UNEx-4Pi can generate a sidelobe-free compact 3D focal spot using only one single objective lens, we built a nonlinear laser-scanning single-objective 4Pi microscope incorporating a mirror (Fig. S6). A phase-only SLM was employed to modulate the wavefront of a continuous-wave (CW) 852-nm laser beam, with the SLM plane optically conjugated to the rear focal plane of the objective lens (100×/1.45) via a lens group with a magnification of 1.2×. In accordance with the time-reversal focusing theory, Eq. (8), the system can produce two foci separated by a distance (D) along the optical axis (Fig. 4a).

The recently reported PA nanoparticles can exhibit a huge nonlinear optical response (*N* > 10). For instance, in the system of Yb^3+^/Pr^3+^-codoped PA nanoparticles, an 852-nm CW laser beam can initiate an optical nonlinear response of up to 46th-order^41^. Here, 18-nm Yb^3+^/Pr^3+^-codoped PA nanoparticles were prepared and employed to demonstrate the proposed UNEx-4Pi concept (Fig. S7). In the experiments, the SLM was used to modulate the excitation beam, enabling the acquisition of 3D images of a single PA nanoparticle. As expected, a series of fluorescence images of two focal spots with varying distances D were obtained by adjusting the parameter *r*_*0*_ according to Eq. (8) (Fig. 4b). The experimental data were in good agreement with the simulations. The distances D were determined through a Gaussian fit, which yielded a value equal to 2*r*_*0*_, in accordance with the theoretical value (Fig. 4c). Furthermore, a robust linear relationship between the parameter *r*_*0*_ and D was maintained over a wide range of working distances, up to 24 µm. Inserting a mirror at the center of the two foci enables an axial dynamic scanning range of 12 µm, which is sufficient to scan across adherently growing mammal cells in life science studies^48,49^.

To verify that the UNEx-4Pi has consistent and superior performance across the entire scanning space, 3D images of individual PA nanoparticles at varying heights from the mirror were captured, and the corresponding PSF intensity profiles were plotted. Since the nonlinearity of PA nanoparticles varies with excitation power, linear 4Pi and UNEx-4Pi images can be obtained using the home-built single-objective 4Pi microscopy at high and low laser intensities, respectively. As shown in Fig. 4d, the UNEx-4Pi nanoscopy efficiently eliminated severe sidelobes and compressed the PSF with the ultrahigh-order optical nonlinearity across the axial scanning range. It is also noteworthy that UNEx-4Pi is able to achieve 3D imaging by just altering the SLM pattern, with no need of axially moving the sample stage as required in conventional 4Pi microscopy, thereby further enhancing system stability. Coupled with non-photobleaching and low-power excitation properties of PA probe, UNEx-4Pi is particularly well-suited for 3D super-resolution imaging and long-term monitoring (Fig. S5).

### 3D super-resolution imaging of single-beam, single-objective UNEx-4Pi

Firstly, the excitation-emission-dependent curves of PA nanoparticles were measured, exhibiting a typical S-shaped profile with a giant optical nonlinearity up to 35th-order in the 450–700 nm emission range and a PA threshold at 65 kW cm^−^^2^ (Fig. S7). Ultrahigh optical nonlinearity can significantly compress the effective PSF, whether in common LSM mode or 4Pi configuration. In contrast, the introduction of a silver mirror in UNEx-4Pi serves to reflect the forward-emitting fluorescence back on the objective lens, almost doubling the collected signal intensity and thus enhancing the image SNR (Fig. S8).

**Fig. 5:**
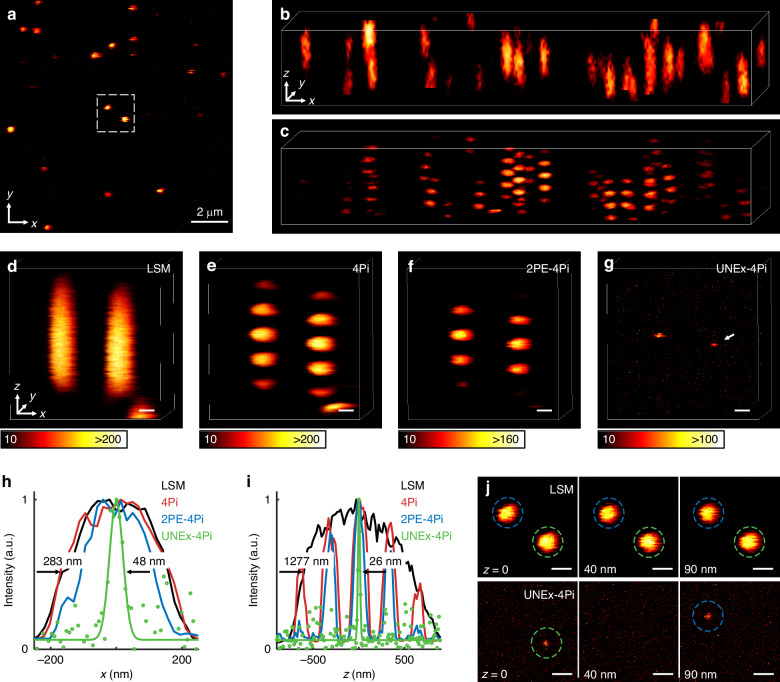
The sidelobe-free UNEx-4Pi super-resolution microscopy. **a** Fluorescence image of PA nanoparticles on the mirror. **b** 3D image of PA nanoparticles in LSM mode. **c** 3D image of PA nanoparticles in 4Pi mode. **d**–**g** 3D images in LSM, 4Pi, 2PE-4Pi, and UNEx-4Pi modes respectively. Scale bars: 200 nm, pixel dwell time: 100 μs, pixel size: 10 nm. **h**, **i** The lateral and axial intensity profiles of the nanoparticle marked by a white arrow in (**g**). The green dots represent the experimental values. The green curves are the fitting curves. The lateral and axial FWHMs of UNEx-4Pi were 48 nm and 26 nm, corresponding to 283 nm and 1277 nm in LSM, respectively. **j** *xy*-slices (z = 0, 40, 90 nm) showed UNEx-4Pi had superior super-resolved optical sectioning capability, compared with LSM. Scale bars: 400 nm, pixel dwell time: 100 μs, pixel size: 10 nm

To showcase the performance of UNEx-4Pi, the Yb^3+^/Pr^3+^-codoped PA nanoparticles distributed on the mirror were imaged (Fig. 5a). For a comparison, the LSM image could be obtained by setting the SLM to a fixed bifocal phase and utilizing a single focus to scan the sample axially by the stage (Fig. 5b). The 4Pi image was achieved by altering the pattern of the SLM, and the severe sidelobes caused by the constructive interference degraded the imaging contrast and resulted in an amount of artifacts (Fig. 5c). As a striking comparison, two individual nanoparticles were imaged respectively under LSM, 4Pi, 2PE-4Pi, and UNEx-4Pi modes, aligning well with the simulation results (Figs. 5d–g and 2a). The linear 4Pi, 2PE-4Pi, and UNEx-4Pi modes were achieved by adjusting the excitation intensity, respectively. As the excitation intensity was decreased to near the PA threshold (68 kW cm^−^^2^), UNEx-4Pi nanoscopy exhibited a lateral resolution of 48 nm and an axial resolution of up to 26 nm (λ/33) (Fig. 5h, i). Remarkably, the axial resolution of UNEx-4Pi was approximately 50 times better than that of LSM mode. As shown in Fig. 5j, these two adjacent nanoparticles with a 90-nm axial spacing could be resolved in sequence, highlighting the superior optical sectioning capability of UNEx-4Pi. In contrast, such two nanoparticles remained indistinguishable in the axial direction in the diffraction-limited LSM imaging.

### Subcellular labeling and UNEx-4Pi imaging

**Fig. 6:**
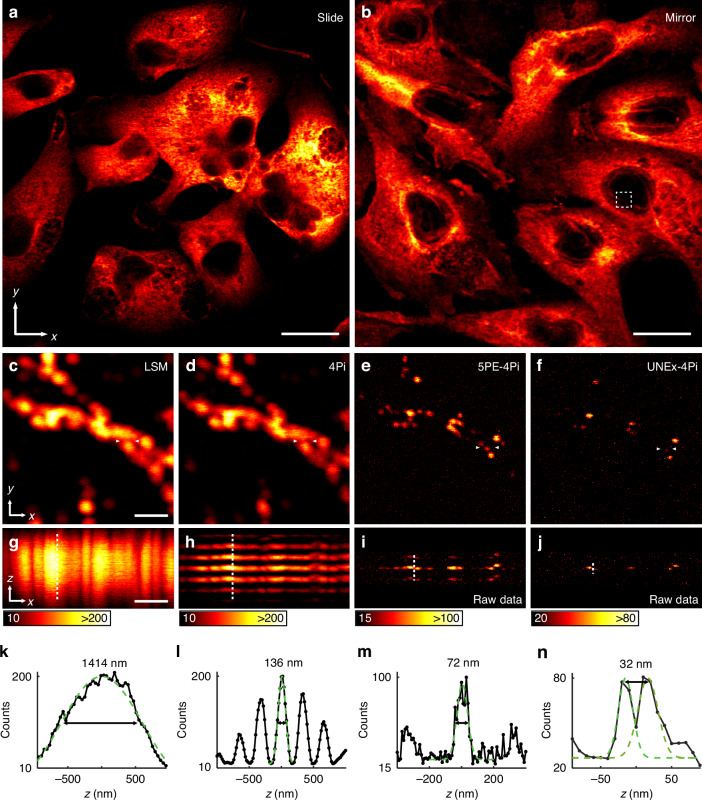
UNEx-4Pi nanoscopy for subcellular super-resolution imaging. **a**, **b** Fluorescence images of BSC-1 cells labeled with PA nanoparticles. Scale bars: 20 μm. The white dashed-box region containing nuclear envelope was enlarged for further comparison, corresponding to magnified images in (**c–j**). **c–f** The maximum intensity projection (horizontal plane) in LSM, 4Pi, 5PE-4Pi, and UNEx-4Pi modes respectively. **g–j** The corresponding intensity projection (vertical plane) in (**c–f**). **k–n** Intensity profiles indicated by the white dash line in (**g–j**). The black dots represent the experimental values, and the green dash lines are the corresponding fitting curves. Scale bars: 1 μm, pixel dwell time: 100 μs, pixel size: 10 nm in **(c–j**)

As a demonstration of the potential applications, we have presented the capabilities of UNEx-4Pi in 3D super-resolution bioimaging. Lanthanide-doped UCNP nanoprobes have been emerging as a unique type of luminescent markers for bioapplications, such as in vitro cellular/subcellular imaging^41,50^, in vivo molecular imaging^51,52^, infrared vision^53^, and optogenetics^54^. Particularly, the subcellular labeling of the emerging lanthanide-doped nanoparticles is highly important, intriguing, but also challenging^55^. The substandard solubility and stability of UCNP nanoprobes in cellular environments have limited their performance in subcellular staining and imaging, resulting in nanoparticles agglomerating or merely attached to the cell surface^56^. The chemical modification of nanoparticle surfaces is vital for improving the intracellular uptake of functional nanoparticles as well as enhancing the labeling efficacy. In this work, we performed surface modification for the PA nanoparticles with DSPE-PEG to further enhance the water solubility and chemical stability. With the optimization of surface modification, the stability and water solubility of UCNPs have been improved (Fig. S9), thus enabling high-quality, large field-of-view cellular staining and imaging (Fig. S10). The cytoplasm of BSC-1 cells labeled with PA nanoparticles were clearly observed (Fig. 6a), demonstrating efficient intracellular uptake and high labeling efficacy of the modified lanthanides-doped nanoparticles. The presence of the silica layer enables cells to grow normally on the mirror surface, exhibiting very similar performance of cellular labeling to that observed on the cover slide (Fig. 6b). An enlarged region of interest from the mirror-fixed sample provided a detailed view of the nuclear envelope for further comparison (Fig. 6c–f). The extracted line profiles of different modes were fitted with Gaussian functions and demonstrated the excellent imaging performance of UNEx-4Pi with a lateral FWHM of 60 nm at an excitation of 72 kW cm^−2^, which is fivefold higher than that of the conventional diffraction-limited mode (Fig. S11). The intensity projection along the *y*-axis revealed that UNEx-4Pi was capable of resolving structures at intervals of 32 nm in the axial direction with no need of any mathematical deconvolution post-processing (Fig. 6j, n). Similarly, UNEx-4Pi easily resolves 40 nm intervals in the axial direction for relatively dense cellular structures (Fig. S12). In an obvious contrast, the LSM mode exhibited poor axial resolution and 4Pi mode was severely constrained by significant sidelobes, while low-order nonlinearity proved inadequate for fully eliminating sidelobes or resolving the fine structure (Fig. 6g–i and k–m). It is reassuring to believe that with the relentless efforts, further progress will be made in subcellular labeling of the emerging lanthanide-doped nanoparticles and, ultimately, in UNEx-4Pi super-resolution microscopy. With further experiments, it was found that the cytotoxicity of PA nanoprobes can be negligible (Fig. S13), and also no significant photodamage to cells was observed under an excitation intensity of 70 kW cm^−2^ during two-hour live-cell imaging (Fig. S14). These facts suggest that UNEx-4Pi have great potential as an indispensable tool for long-term live-cell super-resolution monitoring.

## Discussion

In summary, a deterministic 3D nanoscopy of UNEx-4Pi was proposed and the effectiveness of this method was confirmed both theoretically and experimentally. It was demonstrated that in UNEx-4Pi the main peak was sharpened and its sidelobes were significantly suppressed when increasing the optical nonlinearity. The stubborn sidelobe problem in interference illumination has been for the first time completely eliminated in real physical space. The sidelobe-free, constringent PSF enabled by UNEx-4Pi obviated the necessity for pinhole-based confocal detection and maximized the fluorescence signal collection. In comparison with isoSTED and other 4Pi-derived technologies, UNEx-4Pi enables single-objective 4Pi illumination through a significantly simplified optical setup and a silver mirror. Other than compressing the PSF with the giant nonlinearity, UNEx-4Pi uniquely overcomes the large restrictions of single-objective imaging systems, where the axial resolution is far inferior to the lateral one. The obtained 26-nm (λ/33) axial resolution was nearly 50 times better than that of the conventional LSM. Notably, compared to dual-objective 4Pi configuration, the bifocal refocusing in single-objective 4Pi would introduce spherical aberrations^57^. The applicable sample thickness would be about 12 μm considering that high-order nonlinearity can mitigate these aberrations. The integration of adaptive optics offers promising to extend the UNEx-4Pi imaging depth^58^. A 100 μs pixel dwell time was used for UNEx-4Pi image acquisitions, and the further study of faster PA temporal responses would be favorable for high-speed UNEx-4Pi.

Critically important for practical applications, the facile UNEx-4Pi 3D nanoscopy, which relies on a single objective and a single low-power CW-beam, is highly cost-effective, robust, stable and easy to implement in terms of optical architectures, and it can be readily compatible to the well-commercialized raster or parallel laser-scanning microscope. In addition, we have demonstrated that PA nanoprobes exhibit excellent water solubility and biocompatibility with biological samples. As a demonstration, super-resolution imaging for nuclear envelope of BSC-1 cells was implemented, achieving an axial resolution of 32 nm. PA nanoprobes exhibit unique advantages, including exceptional optical nonlinearity, near-infrared excitation and resistance to photobleaching, which make them ideal for long-term, low-phototoxicity, super-resolution microscopic imaging. Recent advancements in the functionalization and bioconjugation of UCNPs have further expanded their applications such as subcellular organelles targeting and temperature mapping^59^, functional cancer imaging^60^ and observing highly-spatiotemporal biological events^61^. Besides, the uniqueness and merits of deterministic 3D super-resolution strategy can enable wide-ranging nanoscopic applications beyond imaging. For example, the STED technique has been demonstrated for super-resolution lithography^62^, and nonlinear excitation effect has also been explored to achieve 3D printing^4^ and ligand-assisted direct lithography^63^. Therefore, the proposed UNEx-4Pi strategy will provide new insights into achieving sidelobe-free diffraction-unlimited 3D focusing, facilitating advances in frontier areas such as single-beam-driven diffraction-unlimited optical sensing^64^, laser lithography, and optical data storage^65^.

## Material and methods

### Optical setup for UNEx-4Pi nanoscopy

As illustrated in Fig. S6, we built a nonlinear laser-scanning single-objective 4Pi microscope based on a conventional raster laser-scanning microscope (FV1000MPE-S with motorized inverted IX81, Olympus). The 852-nm CW laser was generated by a wavelength-tunable Ti:sapphire laser (Mira HP, Coherent) with the photon energy matching the excited-state absorption for excitation of lanthanide emitters. An 850-nm band-pass filter (FF01-850/10-25, Semrock) was used to purify the other wavelength components of the excitation laser. Then, the beam passed through a pair of achromatic lenses L1, L2 (Thorlabs, AC254-030-A-ML/AC254-150-A-ML) and expanded the beam fivefold to completely cover the SLM (LETO, Holoeye) target plane. The SLM-modulated beam was conjugated to the surface of the galvanometer scanner by a pair of achromatic lenses with focal lengths of 300 mm and 100 mm to ensure minimal distortion during the scanning process. Combined with the scan lens (L5) and the tube lens (L6) of microscope, the SLM target plane was finally enlarged by 1.2 times and conjugated to the rear focal plane of the objective lens (UPLXAPO, 100×/1.45, Olympus). By using a circular SLM area with a radius of 330 pixels to exactly fill the entrance pupil of the objective lens and loading a time-reversal function in this area, we were able to obtain two diffraction-limited focal spots with adjustable distance along the optical axis near the focal plane of the objective lens^28,47^. Subsequently, a mirror for specular reflection was placed at the center of the two foci to induce the interference, forming an axially compressed 4Pi spot. Combined with PA nanoprobes featuring ultrahigh-order optical nonlinearity, this configuration realized an extremely constringent fluorescence excitation spot without any observed sidelobe. In this device, transverse scanning was achieved by controlling the X-Y galvanometric scanners, while axial scanning was achieved by changing the *r*_*0*_ parameter in the phase modulation function loaded by SLM.

The fluorescence signal was collected by the same objective and the visible emission (400–700 nm) was filtered out by the dichroic mirror. A short-pass filter (F2, FF02-694/SP-25, Semrock) was used to further filter the signal and a photomultiplier tube was employed for the imaging study.

### Spectra measurements and laser power-dependent emission intensity

The optical system for spectra measurements shared a similarly optical configuration as the optical system for imaging study. The fluorescence signal was collected by a visible spectrometer (QE65000, 350–1000 nm, Ocean Optics) for further analysis. An optical power meter (PM100D, Thorlabs) with microscope slide power meter sensor head (S170C, Si, Thorlabs) was used to determine the laser power. The diameter of the laser focal spot was defined as the full width at 1/e^2^ of maximum, and the laser intensity of PA excitation was calculated by dividing the power by the area of the laser spot.

### Synthesis of NaYF_4_:Yb/Pr(15/0.5%)@NaYF_4_ nanoparticles

The designed lanthanide-doped nanoparticles were synthesized following previously reported protocols with some modifications^41,66,67^. In a typical synthesis, 5 mL of Ln(CH_3_CO_2_)_3_ (Ln = Y/Yb/Pr) aqueous solution was placed into a 100 mL round-bottom flask, followed by the addition of 7.5 mL of OA and 17.5 mL of ODE. The mixture was heated to 150 °C with stirring for 50 min to form the lanthanide-oleate precursor solution, and then cooled to room temperature. Subsequently, 0.148 g of NH_4_F and 0.761 g of sodium oleate were added to the flask. The mixture was then heated to 40 °C and maintained at that temperature for at least 4 h, before being heated to 110 °C under vacuum to remove methanol. After the methanol was evaporated, the mixture was heated to 270 °C and incubated at that temperature for 1.5 h under an argon atmosphere, and then cooled to room temperature. The nanoparticles were precipitated by the addition of ethanol, followed by centrifugation at 7500 r.p.m. (relative centrifuge force = 4788 g) for 5 min. The obtained nanoparticles were washed several times using ethanol and cyclohexane, and finally re-dispersed in 9 mL of cyclohexane for subsequent use. By controlling the concentration of Yb^3+^, Pr^3+^, and Y^3+^, bare core nanoparticles NaYF_4_:Yb/Pr(15/0.5%) were obtained.

Another 5 mL of Ln(CH_3_CO_2_)_3_ (Ln = Y) aqueous solution with 1 mmol of Y^3+^ was placed into a 100 mL round-bottom flask containing 7.5 mL of OA and 17.5 mL of ODE. The solution was heated to 120 °C for 10 min to remove water, and then further heated to 150 °C for 40 min to form the lanthanide oleate precursor. Subsequently, the solution was cooled to 80 °C. A 3 mL suspension of bare core nanoparticles NaYF_4_:Yb/Pr(15/0.5%) was pipetted into the flask and maintained at this temperature for 30 min to remove cyclohexane. Afterwards, the solution was cooled to 40 °C and maintained at that temperature for at least 4 h, before being heated to 110 °C under vacuum to remove methanol. After the methanol was evaporated, the mixture was heated to 300 °C and incubated at that temperature for 1.5 h; the subsequent steps were the same as for the synthesis of the bare core nanoparticles NaYF_4_:Yb/Pr(15/0.5%). Transmission electron microscopy (TEM) characterization revealed that the diameter of the synthesized nanoparticles NaYF_4_:Yb/Pr(15/0.5%)@NaYF_4_ was 17.9 nm (Fig. S7).

### Preparation of nanoparticle-covered silver mirrors for spectroscopic measurements

To perform spectroscopic and emission intensity characterizations, the sample were prepared by drop-casting the as-prepared nanoparticles (10 μL, 200 μg mL^−1^) onto a silver mirror. After air-drying, the silver mirror was placed on a clean cover glass (thickness no. 1.5H, 170 ± 5 μm, Deckgläser). The sample was kept at room temperature for another 10 h to ensure complete drying before measurement.

### Sample preparations for single-nanoparticle imaging

To image single nanoparticles at varying distances from the mirror surface, PA nanoparticles were incorporated into the polydimethylsiloxane (PDMS) solution and subsequently spin-coated onto a silver mirror (Thorlabs, PF10-03-P01, Reflectance (Average): >97.5% for 450 nm −2 µm, Surface Flatness (Peak to Valley): λ/10@633 nm. Specifically, 1 mL of PA nanoparticles (200 μg mL^−1^) was blended with 1 mL of prepared PDMS solution and sonicated for 15 minutes to ensure uniform dispersion. The resulting mixture was then spin-coated (4000 r.p.m.) onto the silver mirror and cured at 125 °C for 20 minutes to solidify the PDMS. Once the mirror reached room temperature, it was placed on a clean cover glass and a small drop of embedding medium was applied and spread. Any air bubbles were removed, and the cover glass was sealed with nail polish. This process yielded a PDMS colloid layer approximately 15 μm thick, uniformly embedded with PA nanoparticles, on the mirror surface.

### Surface modified for the PA nanoparticles with DSPE-PEG

To improve water solubility and stability, the surface of PA nanoparticles was modified with DSPE-PEG. 8 mL of DCM and 20 mg of DSPE-PEG (2000) were added to 2 mL of DCM containing 0.1 mmol of as-prepared nanoparticles in a 25 mL round flask. After stirring for 2 h at room temperature, DCM was removed by heating and stirring evaporation. The obtained film was hydrated with 10 mL deionized water. The mixture was transferred to a centrifugal tube and purified PANs@DSPE-PEG by ultracentrifugation (14,000 rpm, 30 min). Finally, the @DSPE-PEG was redispersed in 5 mL PBS.

### Cellular sample preparation

The BSC-1 cells (African Green monkey kidney cells) were obtained from the American Type Culture Collection and cultured in Medium DMEM containing 10% fetal bovine serum, 100 U mL^−^^1^ penicillin 100 µg mL^−^^1^ streptomycin in a humidified incubator at 37 °C and 5% carbon dioxide. Cells were passaged early and cryopreserved and maintained in culture for <3 months, treated regularly for mycoplasma contamination using the luciferase mycoplasma detection kit. The BSC-1 cells (15,000 cells/well) were incubated overnight on mirror. The next day, the cells were washed with PBS buffer and fixed at room temperature using 4% PFA for 15 min. Cells were permeabilized in 0.25% Triton X-100 for 15 min at room temperature and were rinsed again using PBS. 5% BSA was then used to block the cells for 30 min at room temperature. When the procedure of blocking was completed, the prepared 200 µg mL^−^^1^ PA nanoprobe were added and incubated at 4 °C overnight. Finally, the cells were then rinsed with buffer three times for subsequent imaging using the multiphoton LSM.

### Characterization methods

Transmission Electron Microscope (TEM) images of nanoparticles were obtained from a transmission electron microscope (JEM-2100 HR, 200 kV, JEOL). The samples were diluted and dispersed in cyclohexane, and then dropped onto the surface of copper grids for TEM test.

## Supplementary information

Supplementary Information

## Data Availability

The source data sets and analyzed during the current study are available from the corresponding authors upon reasonable request.

## Code availability

The codes for theoretical modelling and numerical simulations are available from the corresponding authors on reasonable request.
