# Supplementary material for: Sidelobe-free deterministic 3D nanoscopy with λ/33 axial resolution
Source: Light Sci Appl. 2025 Apr 21;14:168. doi: 10.1038/s41377-025-01833-x (PMC12012212; doi:10.1038/s41377-025-01833-x)
Supplement: Supplementary file 1 — Supplementary Information [file 41377_2025_1833_MOESM1_ESM.pdf]

Supplementary Information for

Sidelobe-free deterministic 3D nanoscopy with λ/33 axial resolution

Binxiong Pan, Baoju Wang, Yue Ni, Qi Zhao, Yuqi Wang, Yuyan Cai, Qiuqiang Zhan

TABLE OF CONTENTS

1. Supplementary Figures 1-14

2. Supplementary Table 1

3. References

Supplementary Figures:

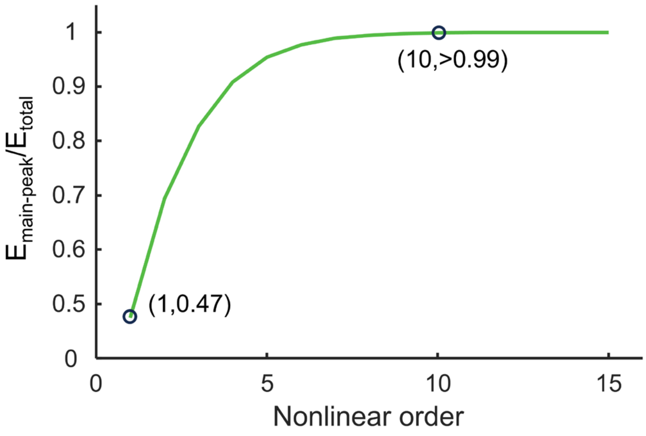

Fig. S1 Energy ratio versus nonlinearity order. The sidelobes of the 4Pi focus can be thoroughly eliminated when the nonlinearity order *N* reaches 10.

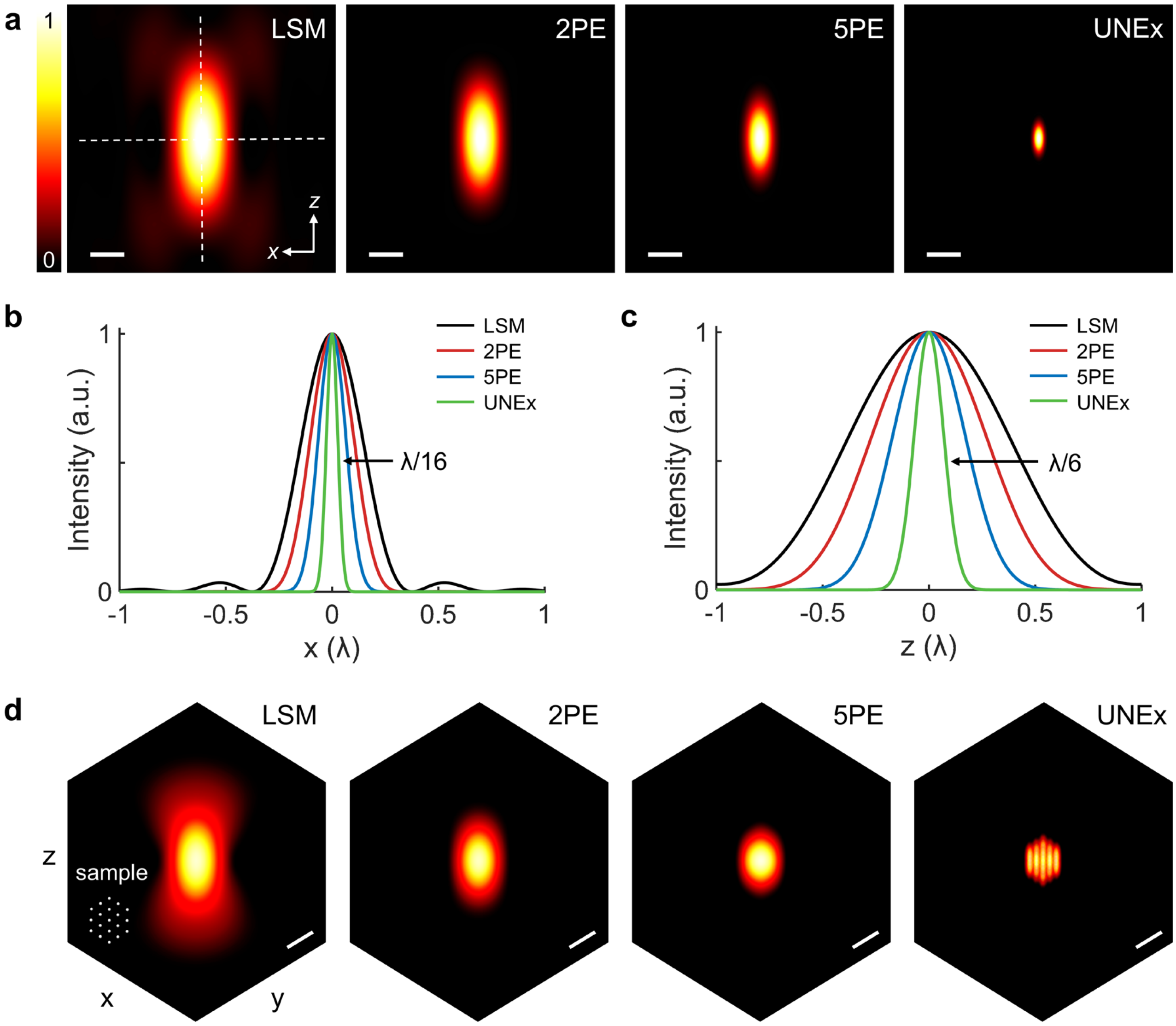

Fig. S2 The simulated PSFs or images for nonlinear excitation LSM. a The *xz*-sections of the intensity distributions in LSM, 2PE-LSM, 5PE-LSM and UNEx-LSM excitation modes. Scale bar: λ/4. λ is the excitation wavelength, NA of the objective lens is 1.45. **b** and **c** showed the lateral and axial intensity profiles, corresponding the white dashed lines in **a**. The lateral FWHM of UNEx-LSM is λ/16, and the axial FWHM is λ/6. **d** The simulated imaging results of different excitation modes for a 3×3×3 sample array with a space interval of 100 nm. Scale bars: 400 nm.

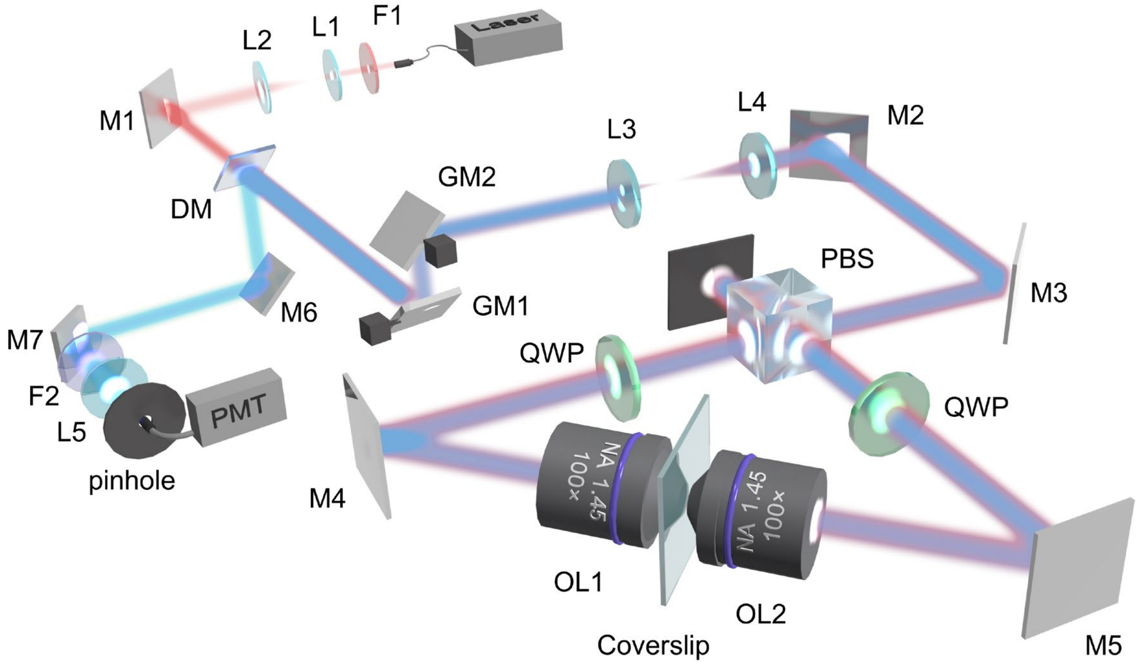

Fig. S3 Schematic diagram of a conventional dual-objective 4Pi microscope. F: filter; L: lens; M: silver mirror; DM: dichroic mirror; GM: galvanometer scanning mirrors; PBS: polarization beam splitter; QWP: quarter-wave plate; OL: objective lens; PMT: photomultiplier tubes.

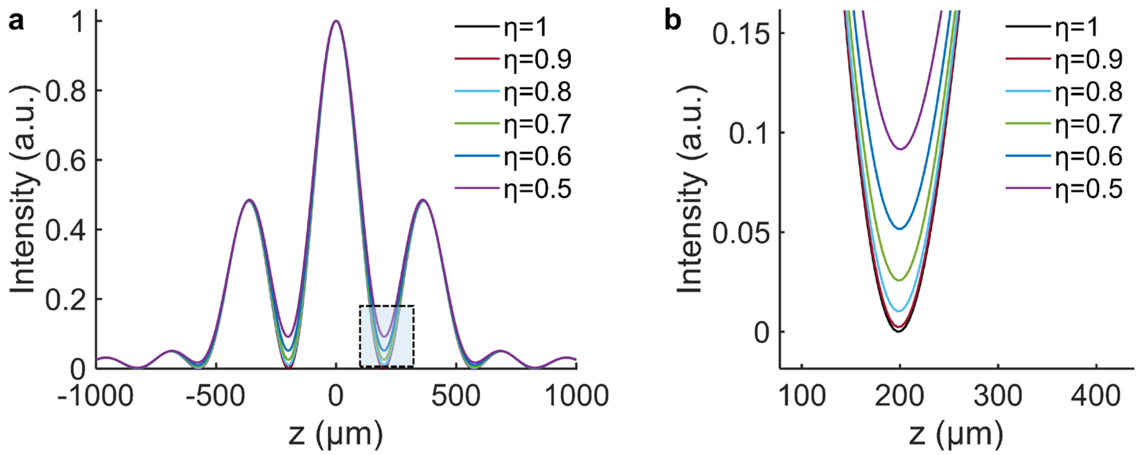

Fig. S4 The simulated impact of the mirror reflectivity on the PSF. a Comparisons of the normalized intensity profiles along the *z*-axis for different reflectance η. **b** The boxed area in **a**. When the reflectivity η = 1, the trough intensity between the main peak and sidelobes approached zero. As the reflectivity decreases, the trough intensity increases accordingly and their boundary gets blurred.

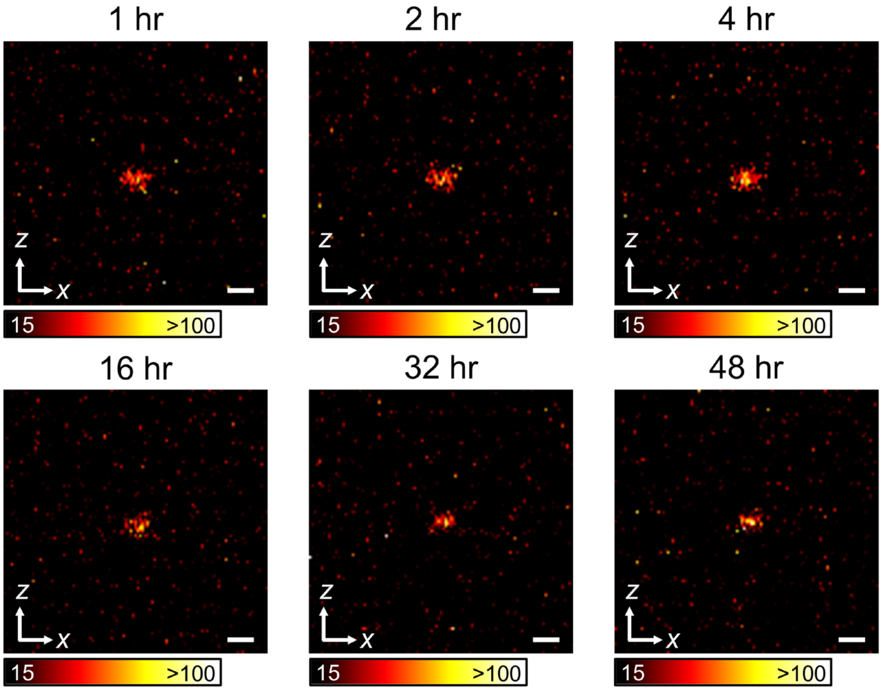

Fig. S5 Long-term stability of UNEx-4Pi nanoscopy (48 hours observation). Super-resolution imaging in *xz* plane was implemented in the UNEx-4Pi nanoscopy and no significant change in resolution was observed over long-term laser scanning (e.g., 48 hours). The non-contact, single-objective, single-beam 3D scanning configuration resulted in high imaging stability, preventing frequent calibration and maintenance. In addition, no photobleaching was observed over 48 hours of laser scanning, which is very important for long-term monitoring. Scale bar: 100 nm, pixel dwell time: 100 μs, pixel size:10 nm.

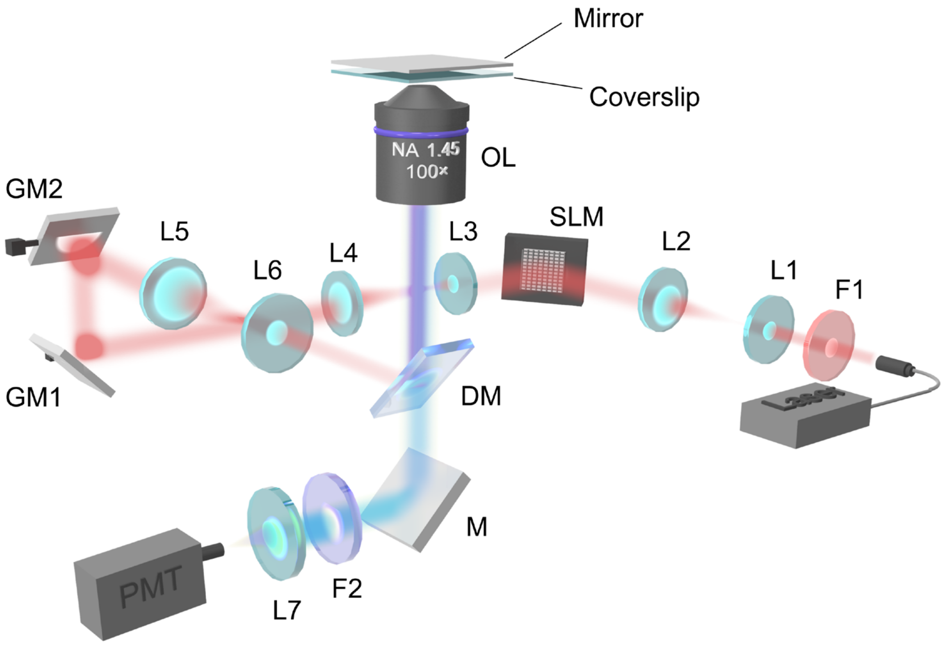

Fig. S6 Schematic diagram of the optical setup employed in this study. F1: 850/10-nm band-pass filter; F2: 694-nm short-pass filter; L1, L2, L3, L4: achromatic lenses with focus length of 30 mm, 150 mm, 300 mm and 100 mm; SLM: spatial light modulator (LETO, Holoeye); GM: galvanometer scanning mirrors; L5, L6: scan lens and tube lens with magnification of 3.6; DM: 690-nm short-pass dichroic mirror; OL: objective lens (UPLXAPO, 100×/1.45, Olympus); PMT: Photomultiplier tubes.

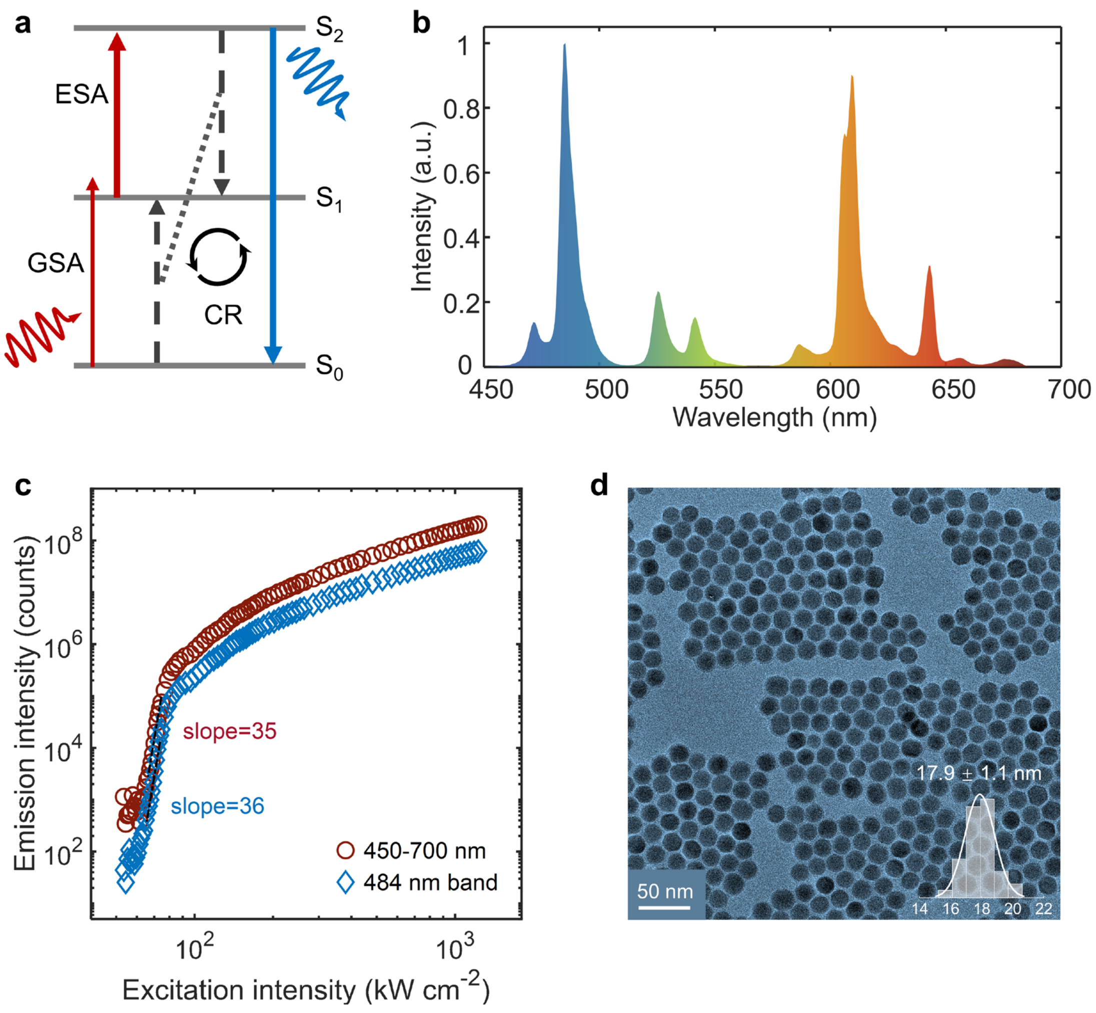

Fig. S7 The PA characterizations for Yb^{3+}/Pr^{3+} co-doped nanoparticles. a Energy transfer mechanism of PA phenomenon. The electrons at the ground state (S_0) are firstly promoted to the metastable state (S_1) through non-resonant ground state absorption (GSA) and followed by the higher-energy excited state (S_2) to be populated via the resonant excited state absorption (ESA). When the excitation intensity exceeds a critical threshold, the ESA and cross-relaxation (CR) process constitute a positive energy-looping cycle, boosting the population of the emitting levels, and leading to a sharp emission burst. **b** Fluorescence emission spectrum of Yb^{3+}/Pr^{3+} co-doped PA nanoparticles. **c** The experimental curves of emission intensity versus excitation intensity for nanoparticles on the reflective mirror. **d** Transmission electron microscopy images and size distributions of the as-synthesized Yb^{3+}/Pr^{3+} PA nanoparticles (average size: 17.9 ± 1.1 nm in diameter).

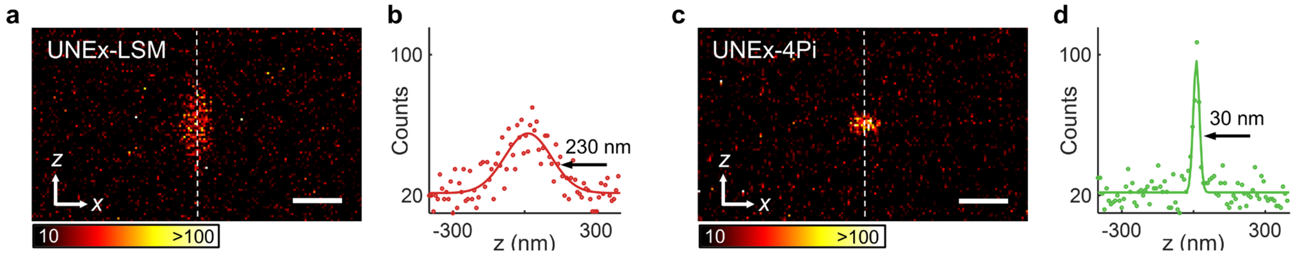

Fig. S8 The comparison of imaging PSFs between the UNEx-LSM and UNEx-4Pi configurations. **a** The *xz*-section of the intensity distributions in the UNEx-LSM configuration. Scale bar: 200 nm, pixel dwell time: 100 µs, pixel size:10 nm. **b** The axial intensity profiles of **a**, and the axial FWHM was 230 nm. **c** The *xz*-section of the intensity distributions in the UNEx-4Pi configuration. Scale bar: 200 nm, pixel dwell time: 100 µs, pixel size:10 nm. **d** The axial intensity profiles of **c**, and the axial FWHM was 30 nm.

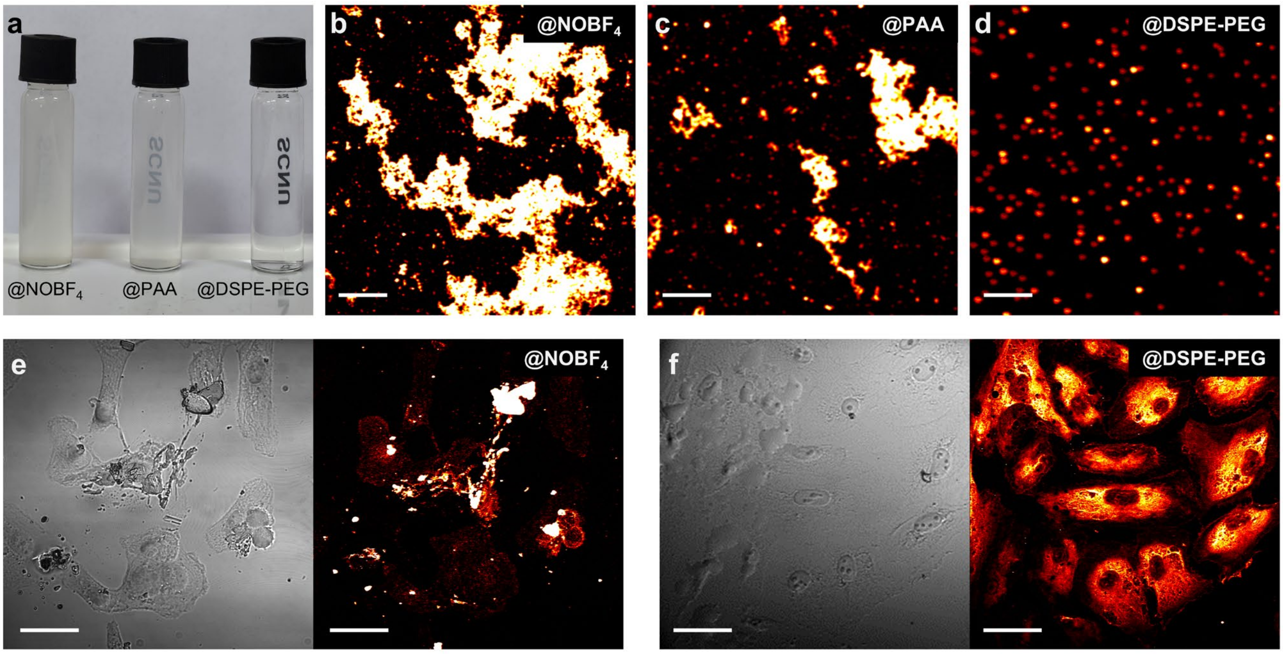

Fig. S9 Surface modification, stability and cellular uptake for the PA nanoparticles. a Water solubility of PA nanoparticles in PBS buffer solution. While nanoprobes@DSPE-PEG exhibited better water solubility compared to nanoprobes@$NOBF_4$ and nanoprobes@PAA. **b-d** Luminescence microscopic imaging of PA nanoparticles with different modifications dispersed in water. At the same concentration, the nanoparticles with different modifications were suspended for luminescence imaging, nanoprobes@DSPE-PEG exhibited excellent particle dispersibility. Scale bars: 10 μm. **e-f** Fluorescence imaging of BSC-1 cells labeled with $NOBF_4$ and DSPE-PEG modified PA nanoparticles. Intracellular labeling with nanoprobes@DSPE-PEG showed much better uniformity compared to nanoprobes@$NOBF_4$. Scale bars: 40 μm.

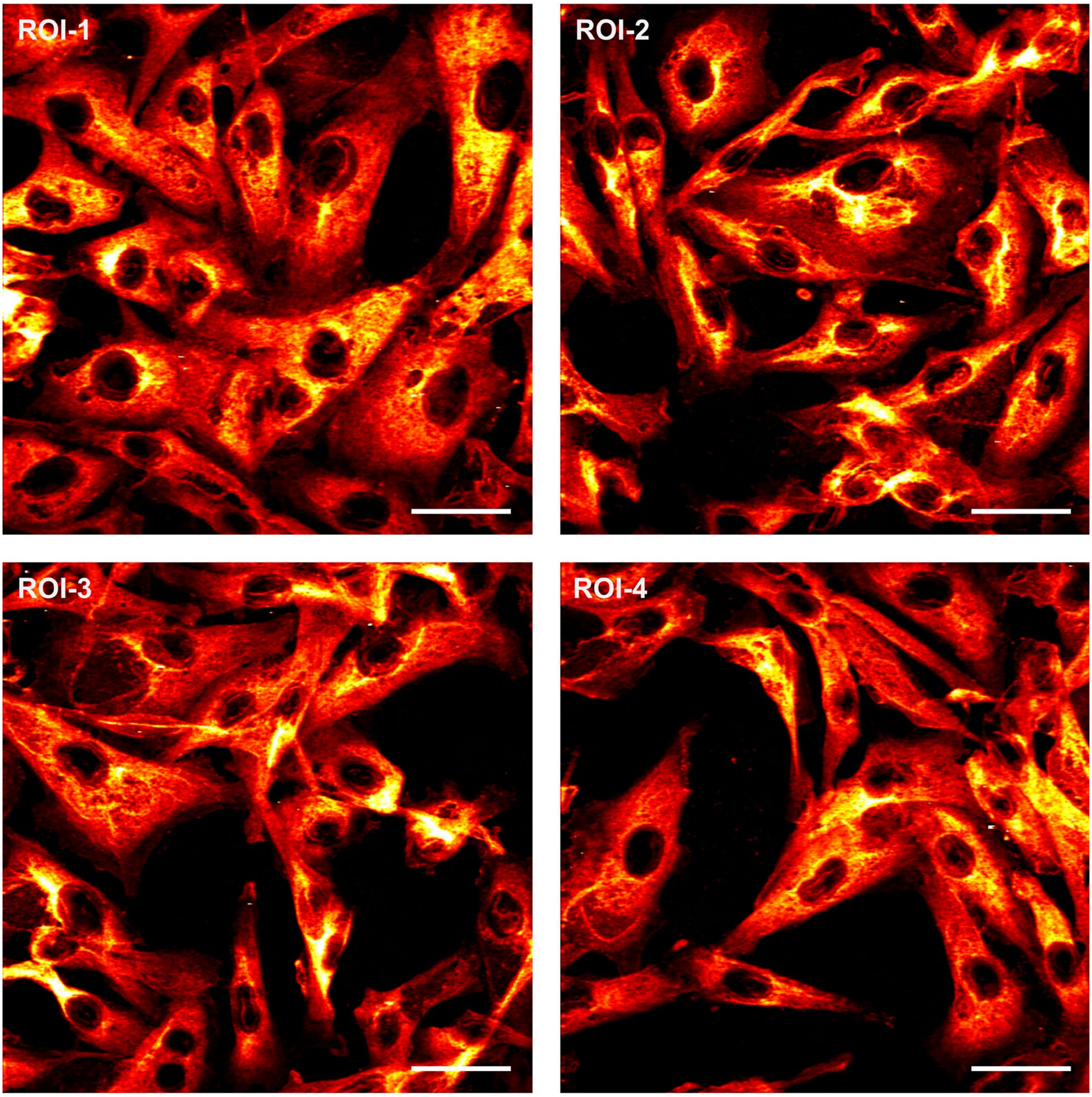

Fig. S10 Large field-of-view imaging of PA nanoprobes stained BSC-1 cells. The microscopic imaging of BSC-1 cell with four different region-of-interest (ROI) showed that the surface modified PA nanoprobes have good intracellular invasiveness and the cytoplasm and nuclear envelope were labeled nicely, also demonstrating the excellent stability and water-solubility of PA nanoparticles. Meanwhile, the issues of nanoparticles agglomerating or merely attached to the cell surface were addressed very well. Scale bars: 40 μm.

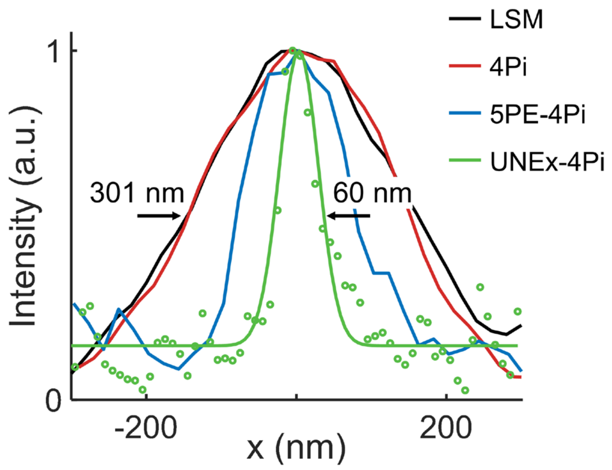

Fig. S11 The lateral intensity profiles of the subcellular structure. The black, red, blue and green lines represented the LSM, 4Pi, 5PE-4Pi and UNEx-4Pi modes, respectively. The green circles represented the experimental values. The green fitting curve showed that the lateral FWHM in UNEx-4Pi mode was 60 nm. For comparison, it was 301 nm in LSM mode.

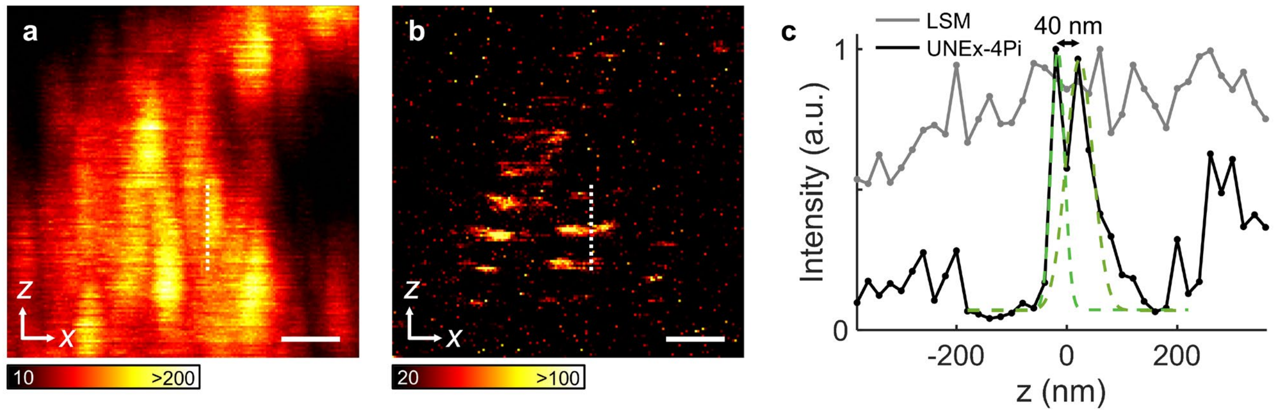

Fig. S12 UNEx-4Pi nanoscopy for relatively dense cellular structures imaging. The max intensity projection along the *y*-axis in LSM (**a**) and UNEx-4Pi (**b**) modes. Scale bars: 500 nm, pixel dwell time: 100 μs, pixel size: 20 nm. **c** Intensity profiles at positions indicated by the white dash line in **a**, **b**.

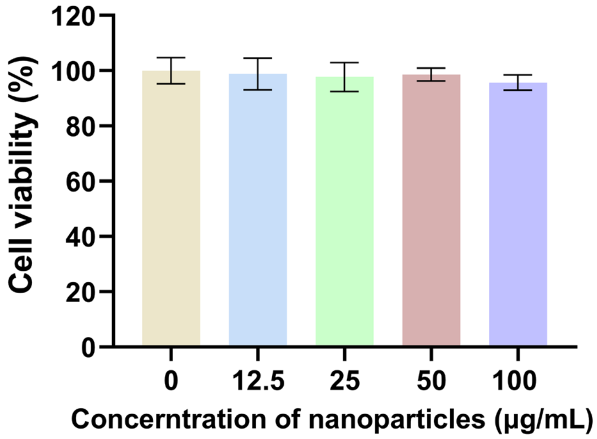

Fig. S13 The cytotoxicity of PA nanoprobes was examined by CCK8 assay in BSC-1 cells. The cytotoxicity of PA nanoprobes at high concentrations of 100 μg mL^{-1} was negligible.

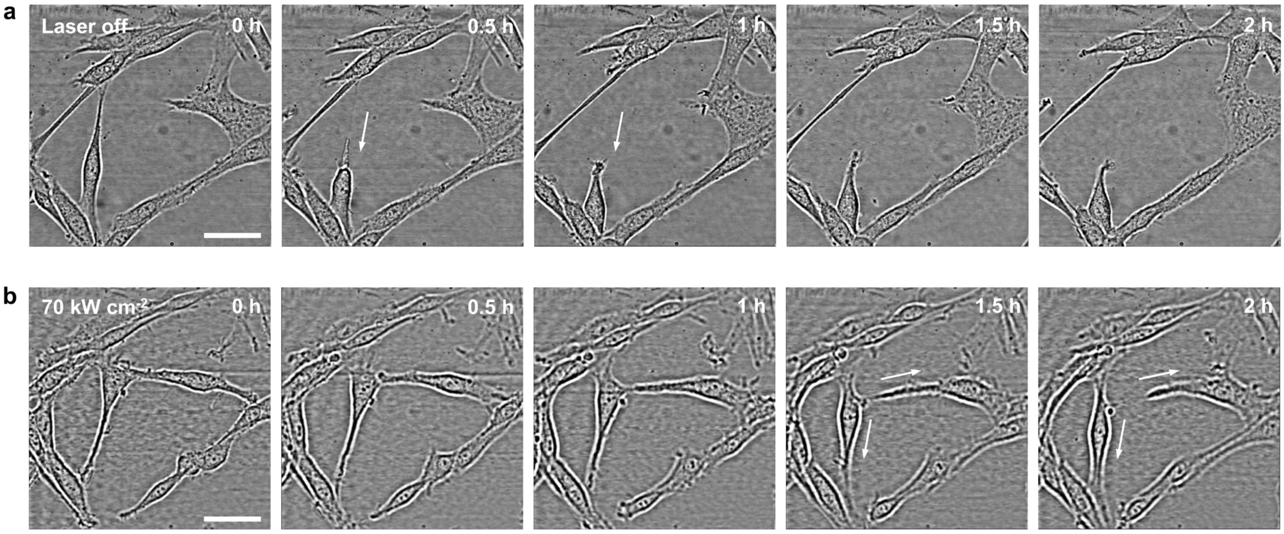

Fig. S14 Phototoxicity test for UNEx-4Pi microscope. Whether the 852-nm laser was turned off (**a**) or was continuously illuminated at 70 kW cm^{-2} (**b**) for 2 hours, the cellular activity remained normal. Scale bars: 30 μm.

Table S1: Technical comparison of 4Pi-related microscopy and UNEx-4Pi nanoscopy

	Type A 4Pi	Type C 4Pi	2PE-4Pi	isoSTED	ISO	UNEx-4Pi
**Number of beams**	2	2 excitation + 2 detection	2	6	Single	**Single**
**Number of objectives**	2	2	2	2	Single	**Single**
**Interference of detection**	No need	Required	No need	No need	No need	**No need**
**Laser intensity**	Moderate	Moderate	Very high	Very high	Moderate	**Low**
**Lateral resolution**	200-300 nm	200-250 nm	300-400 nm	40 nm (λ/13)	200-300 nm	**48 nm (λ/18)**
**Axial resolution**	~140 nm	~100 nm	~140 nm	44 nm (λ/12)	~200 nm	**26 nm (λ/33)**
**Sidelobes intensity [a)]**	50%	20%	30%	10%	50%	**Zero**
**Data reconstruc-tion**	Deconvolution	Deconvolution	Deconvolution	Deconvolution	Deconvolution	**No need, No artifact**
**References**	1,2	2,3	4,5	6,7	8,9	**This work**

[a)] Ratio of the primary sidelobe intensity to main peak intensity. The data in the table are theoretical value. Except for isoSTED, the other 4Pi-derived techniques do not take into account the role of confocal pinholes when calculating the sidelobe. Due to the existence of various aberrations in the experiment, the experimental value is usually higher than the theoretical value.

References

1. Hell, S. & Stelzer, E. H. K. Properties of a 4Pi confocal fluorescence microscope. *Journal of the Optical Society of America A* **9**, 2159-2166 (1992).
2. Pawley, J. B. Handbook of Biological Confocal Microscopy. (New York: Springer, 2006).
3. Gugel, H. et al. Cooperative 4Pi excitation and detection yields sevenfold sharper optical sections in live-cell microscopy. *Biophysical Journal* **87**, 4146-4152 (2004).
4. Hell, S. & Stelzer, E. H. K. Fundamental improvement of resolution with a 4Pi-confocal fluorescence microscope using two-photon excitation. *Optics Communications* **93**, 277-282 (1992).
5. Hänninen, P. et al. Two-photon excitation 4Pi confocal microscope: enhanced axial resolution microscope for biological research. *Applied Physics Letters* **66**, 1698-1700 (1995).
6. Schmidt, R. *et al.* Spherical nanosized focal spot unravels the interior of cells. *Nature Methods* **5**, 539-544 (2008).
7. Hao, X. et al. Review of 4Pi Fluorescence Nanoscopy. *Engineering* **11**, 146-153 (2022).
8. Mudry, E. et al. Isotropic diffraction-limited focusing using a single objective lens. *Physical Review Letters* **105**, 203903 (2010).
9. Le Moal, E. et al. Isotropic single-objective microscopy: theory and experiment. *Journal of the Optical Society of America A* **28**, 1586-1594 (2011).
